# Comparative analysis of the endophytic bacteria inhabiting the phyllosphere of aquatic fern *Azolla* species by high-throughput sequencing

**DOI:** 10.1186/s12866-022-02639-2

**Published:** 2022-10-11

**Authors:** Yan-Qiu Yang, Su-Fang Deng, You-Quan Yang, Zhao-Yang Ying

**Affiliations:** 1 Agricultural Ecology Institute, Fujian Academy of Agricultural Sciences, Fuzhou, China; 2 National Azolla Germplasm Resource Center, Fuzhou, China

**Keywords:** *Azolla*, Phyllosphere, Endophytic bacteria, High-throughput sequencing, Bacterial diversity, Co-evolution

## Abstract

**Background:**

*Azolla* is a small floating fern living in symbiosis with nitrogen-fixing cyanobacteria and provides a variety of important ecosystem benefits. Previous studies have presented that *Azolla* harbors diverse bacteria that may play a key role in host fitness and productivity. However, the characteristics of endophytic bacteria inhabiting the phyllosphere of different species of *Azolla* have not yet been fully understood.

**Results:**

In this study, the 16S ribosomal DNA (rDNA) V5-V7 region of bacteria was determined by Illumina high-throughput sequencing platform to study the diversity and richness of endophytic bacterial communities in the phyllosphere of five *Azolla* species collected from different countries. A total of 1150 operational taxonomic units (OTUs) were detected for the endophytic bacteria community. According to the α diversity indices, the diversity of bacteria was ordered as *Azolla imbricata* > *A. pinnata* > *A. filiculoides* > *A. mexicana* > *A. caroliniana*. The PCoA results displayed that the bacterial communities of *A. mexicana* and *A. caroliniana* shared the highest similarity, followed by the similarity between *A. pinnata* and *A. imbricata*, and they were significantly distinct from the community of *A. filiculoides*. The dominant bacteria of *Azolla* mainly belonged to the phylum of Proteobacteria, followed by Actinobacteria, Chlorobillobacteria, and Firmicutes. In detail, the relative abundance of Proteobacteria in *A. imbricata* was 52.23%, whereas it was more than 80.00% in the other four species of *Azolla*. Notably, *Herbaspirillum* (45.91%, 44.08%) and *Methylophilus* (29.97%, 37.96%) were the main genera inhabiting *A. mexicana* and *A. caroliniana* respectively. *Ferrovibrio* (18.54%) and *Rhizobium* (16.68%) were the dominant genera inhabiting *A. filiculoides*. The group of unidentified genera (41.63%, 44.92%) consisted most of the bacteria in *A. imbricata* and *A. pinnata* respectively. Further analysis suggested that the significant different bacteria identified in LDA Effect Size analysis existed *Azolla* species-specific patterns.

**Conclusions:**

In summary, all results suggested that the diversity and composition of the endophytic bacterial communities were different in *Azolla* species.

**Supplementary Information:**

The online version contains supplementary material available at 10.1186/s12866-022-02639-2.

## Background

Plants share their habitats with a wide variety of microorganisms, including bacteria, archaea, fungi, viruses, and protozoa [1, 2]. Plant hosts and their associated microorganisms are increasingly seen as “holobiont”, whose ecology and evolution are inextricably intertwined [3, 4]. It is well established that bacteria colonize in the rhizosphere and phyllosphere of plants, which contribute to the absorption and utilization of nutrients, improve immunity, and adapt to abiotic /biotic stress, while the specific enrichment of bacteria is influenced by biotic/abiotic factors [5–8]. Previous studies have shown that bacteria inhabiting plants such as maize [9], *Arabidopsis* [10], rice [11], and wheat [12] were subject to complex regulation by plant hosts and bacterial communities [13]. Therefore, a better understanding of the mechanism by which plant genetic factors regulate endophytic bacterial diversity and composition will contribute to plant breeding and open up new avenues for the targeted use of plant-bacteria interactions in agriculture.

*Azolla* is a genus of small floating aquatic ferns comprising about seven to nine species which are globally distributed from tropical to temperate zones in the world, and all species belong to two subgenera, namely the *Euazolla* subgenus including *A. filiculoides*, *A. mexicana* and *A. caroliniana*, *A. microphylla*, *A. rubra*; and the *Rhizosperma* subgenus including *A. pinnata*, *A. nilotica* and *A. imbricata* [14, 15]. Moreover, *Azolla* is remarkable for its massive growing capability which is mainly due to its ability to fix atmospheric nitrogen through their symbiotic cyanobacteria *Nostoc azollae*, which lives within specialized leaf cavities of *Azolla*, and the symbiont performs a variety of significant ecological benefits [16]. For instance, the *Azolla*-*Nostoc azollae* symbiont plays a positive role in increasing rice production and quality in the rice-*Azolla* co-cropping system [17], saving water and nitrogen fertilizer [18], reducing pesticide consumption [19] and greenhouse gas emissions [20]. In addition, *Azolla-*bacteria was used to treat wastewater contaminants for its capacity of heavy metal tolerance and bio-degradation [21]. Hence, *Azolla* combined with bacteria is an important resource for efficient and sustainable agriculture. However, the characterization of phyllosphere endophytic bacteria inhabiting different species of *Azolla* has not yet been fully understood.

As early as the late twentieth century, scientists isolated and identified several phyllosphere endophytic bacteria, such as *Arthrobacter* and *Agrobacterium* from *A. caroliniana* [22, 23]. Since the twenty-first century, scientists detected diverse bacteria living in the leaf cavities of *A. microphylla* which was cultivated under nitrogen deficiency by rDNA PCR, 16S rDNA PCR-DGGE, and T-RFLP methods [24, 25]. Dijkhuizen et al. (2018) [26] analyzed the symbiotic bacterial community of *A. filiculoides* using metagenomic sequencing and revealed that *Nostoc azollae* and *Rhizobium* were the dominant symbiotic bacteria inhabiting the phylloshpere of *A. filiculoides*, which may play a critical determinant role in nitrogen fixation and denitrification. In addition, Chen et al. (2019) [27] revealed that *A. microphylla* harbor salt-resistant Archaea, and this may benefit the plants surviving in a high concentration salt environment. Overall, as Carrapico F. (2002) [28] proposed that bacteria should be seen as a second symbiotic partner with *Azolla*, and the two partners worked closely together with generation and need more attention.

The concern in exploring endophytic bacteria inhabiting the phyllosphere of *Azolla* extends beyond that of detecting potential biodiversity depending on the species, as leaves and stems- borne bacteria are likely to impact vegetative production and development. In this study, we evaluate the phyllosphere endophytic bacterial communities and diversity of five species of *Azolla* collected from different countries by high throughput sequencing. In particular, we investigated the presence of dominant bacteria at different taxa levels inhabiting *Azolla*, aiming to reveal the species-specific bacterial enrichment properties of *Azolla* and providing microbial information for rational use of *Azolla*-bacteria and breeding of *Azolla*.

## Results

### OTU composition of the bacterial community

The high throughput sequencing finally obtained 892,262 high-quality sequences (Clean tags) for subsequent analysis with a high-quality rate of 90.80%. Sequences were clustered at 97% similar levels yielding 1184 OTUs, remaining 1150 after leveling. In addition, to obtain the taxonomic information corresponding to each OTU, the RDP Classifier algorithm was used to annotate OTU at phylum to genus level (Table 1).

**Table 1:** The endophytic bacterial OTU composition of five species of *Azolla*

Sample	Phylum	Class	Order	Family	Genus
Afi	19	37	73	121	156
Ame	14	25	49	83	103
Aca	12	21	41	63	79
Api	19	38	66	125	161
Aim	26	52	95	156	194

**Fig. 1:**
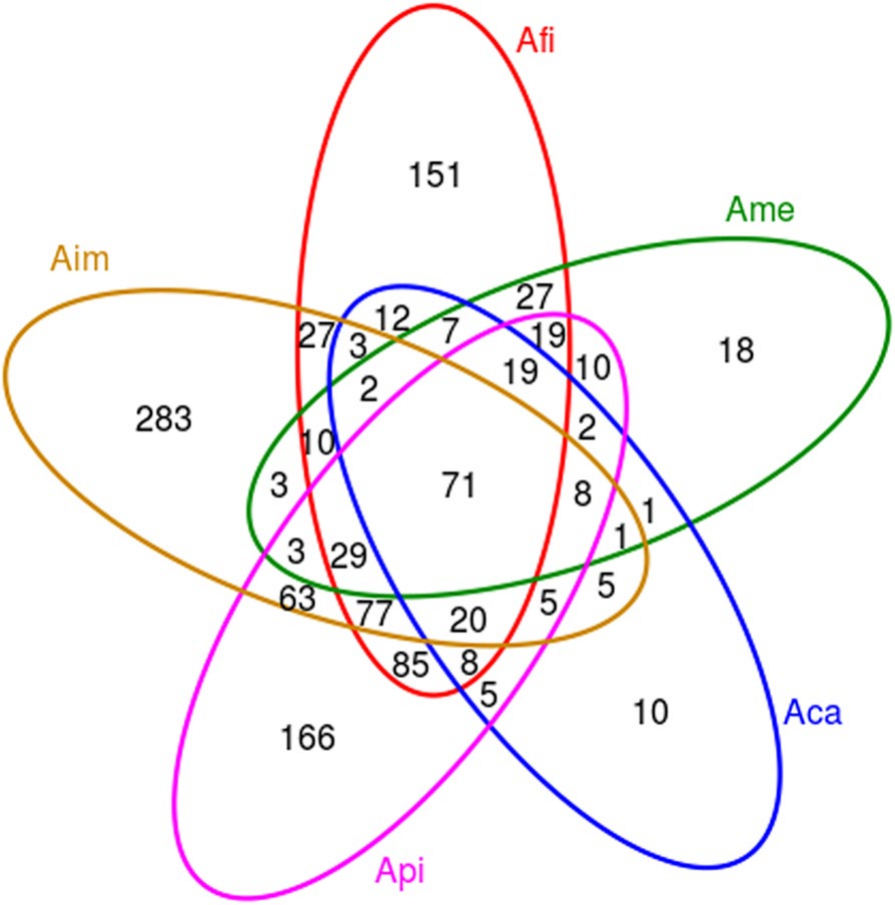
Venn diagram of OTU distribution of endophytic bacterial community detected in five species of *Azolla*. Notes: Different colors represent different samples. The overlapping area of the circles of different colors is the intersection, that is, the same OTU that overlaps several color circles, and the unique OTU relative to the non-overlapping part

To intuitively show the difference of OTU in different *Azolla* species, a Venn diagram showing the distribution of common or unique OTU in five species of *Azolla* was detailed in Fig. 1. At the OTU level, OTU common to the five species of *Azolla* represented 6.17% (71) of the total amount of OTU, and the remaining OTU were cross-shared or independently owned by the samples. Among them, Aim contained the most specific OTU, for 46.39% (283) of its total OTU, indicating the most abundant specific bacterial groups owned by Aim. Furthermore, Afi and Api jointly owned 328 OTU, for 56.07% and 55.59% of their total OTU, respectively, indicating more bacterial taxa shared by both. Aim and Api shared 276 OTU, accounting for 45.25% and 46.78% of their total OTU, respectively. Ame and Aca jointly owned 111 OTU, accounting for 49.78% and 66.07% of their total OTU, respectively, suggesting similarities in these two communities.

### α diversity analysis of bacterial communities

To further assess the abundance and diversity of the endophytic bacterial community in *Azolla*, the α diversity index of samples were calculated and results were detailed in Table 2. The PD whole tree is a diversity index that takes into account the species abundance and evolutionary distance, and the results indicated that the abundance of the bacteria in Afi, Api, Aim was significantly higher than it was in Ame and Aca. Chao1 index was used to estimate the abundance of species in the community, the larger index indicating a higher community richness. Chao1 index indicated the abundance of bacterial communities ordered as Afi > Api > Aim > Ame > Aca. Shannon index was used to estimate bacterial diversity, the larger values indicating higher community diversity. The Shannon index of the bacterial community in Aim was the highest, while the Shannon index of Aca was the lowest. Simpson index was used to estimate bacterial diversity in samples, considering both abundance and uniformity, and greater values indicate higher community diversity. The Simpson index indicated that the bacterial community diversity was ranked as Aim > Api > Afi > Ame > Aca, this result was consistent with the Shannon index, but it was slightly different from the PD whole tree and the Chao1 index.

**Table 2:** Richness and diversity index of endophytic bacteria in five species of *Azolla*^1^

Samples	PD whole tree	Chao1 index	Shannon index	Simpson index
Afi	38.41 ± 2.44^a^	505.14 ± 26.07^a^	4.58 ± 0.13^b^	0.91 ± 0.01^b^
Ame	20.10 ± 5.94^b^	181.82 ± 67.65^b^	2.27 ± 0.14^c^	0.68 ± 0.02^c^
Aca	8.93 ± 2.56^b^	134.05 ± 64.38^c^	1.69 ± 0.08^d^	0.64 ± 0.01^c^
Api	37.41 ± 5.22^a^	431.30 ± 61.61^ab^	4.92 ± 0.10^b^	0.91 ± 0.01^b^
Aim	32.38 ± 3.02^a^	367.25 ± 42.24^b^	6.45 ± 0.06^a^	0.98 ± 0.00^a^

Data are means of triplicates (Mean ± SEM, *n* = 3). Different letters indicate the significant difference between samples (*P* < 0.05)

^1^a, b, c, are different, indicating significant differences between each group. ab and a are compared or ab and b are compared, because both have duplicate letters, indicating no significant difference between each group.

### β diversity analysis of bacterial communities

**Fig. 2:**
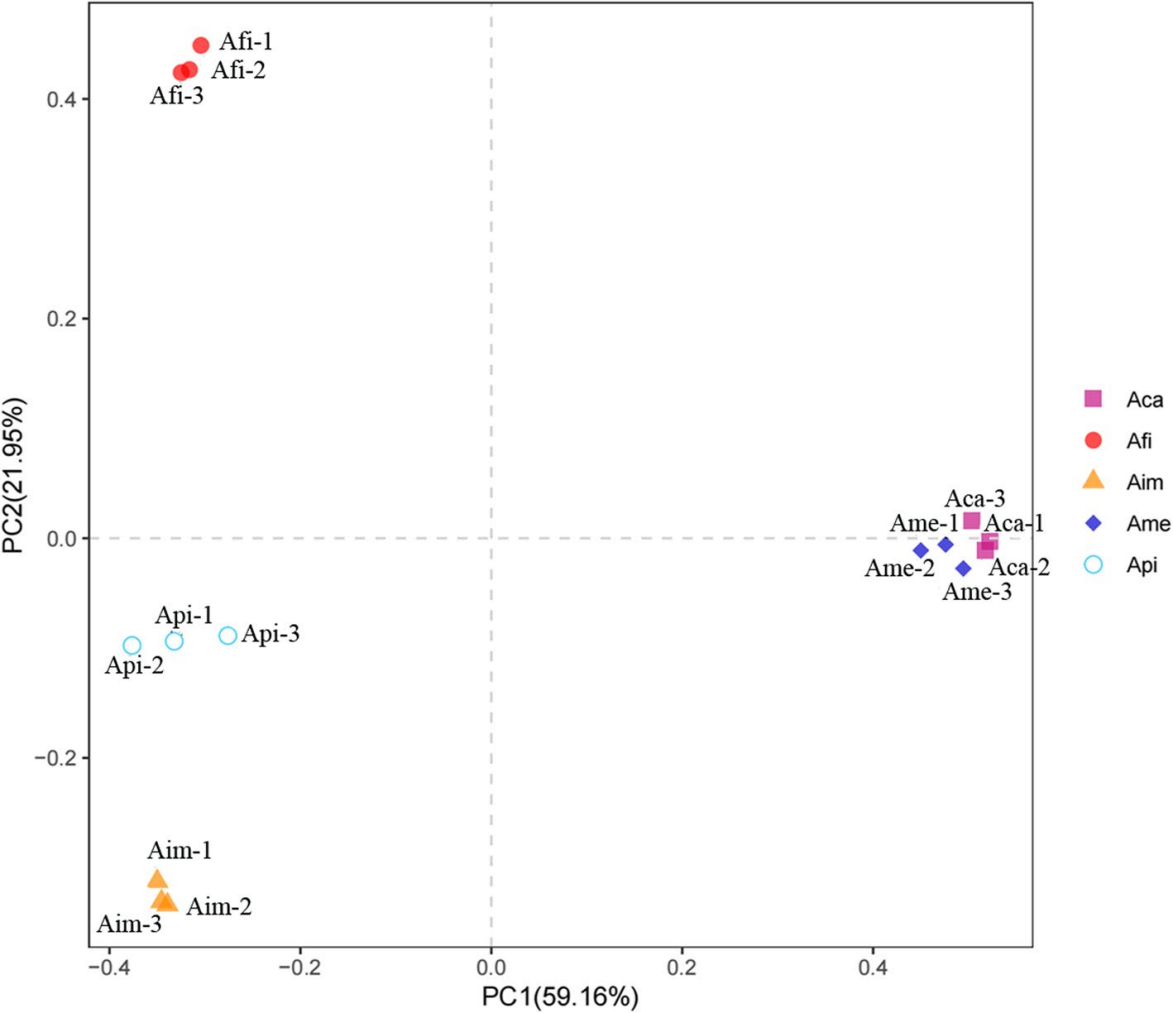
PCoA analysis of endophytic bacterial communities in five species of *Azolla*. Notes: The analysis method is based on Bray Curtis-based principal coordinate analysis (PCoA) using ade4 and ggplot2 packages of R (v3.6.0) software. Points of different colors or shapes represent different grouping situations, and the scale of the horizontal and vertical axes is a relative distance. PC1, PC2 represent the suspected influencing factors of the microbial composition of the two groups, which need to be combined with the sample characteristic information

The bray Curtis-based principal coordinate analysis (PCoA) method was used to investigate the similarities or differences in the community structure in different species of *Azolla*. Bray–Curtis distance considers only the presence of species and their abundance, no evolutionary relationship between species, and distance 0 indicates the complete consistency of the species in two communities. The PCoA of the bacterial community exhibited four distinct clusters of samples (Fig. 2). PCoA 1 and PCoA 2 explained 59.16% and 21.95% of the variation, respectively. The bacterial communities of Ame and Aca were very close to each other, indicating the similarity in their endophytic bacterial communities. The placement of Api and Aim communities was closer, indicating that their species structure was more similar. The distance of Afi from the other four *Azolla* species was the greatest indicating it owned most species-specific bacterial communities.

### Composition analysis of the bacterial community

**Fig. 3:**
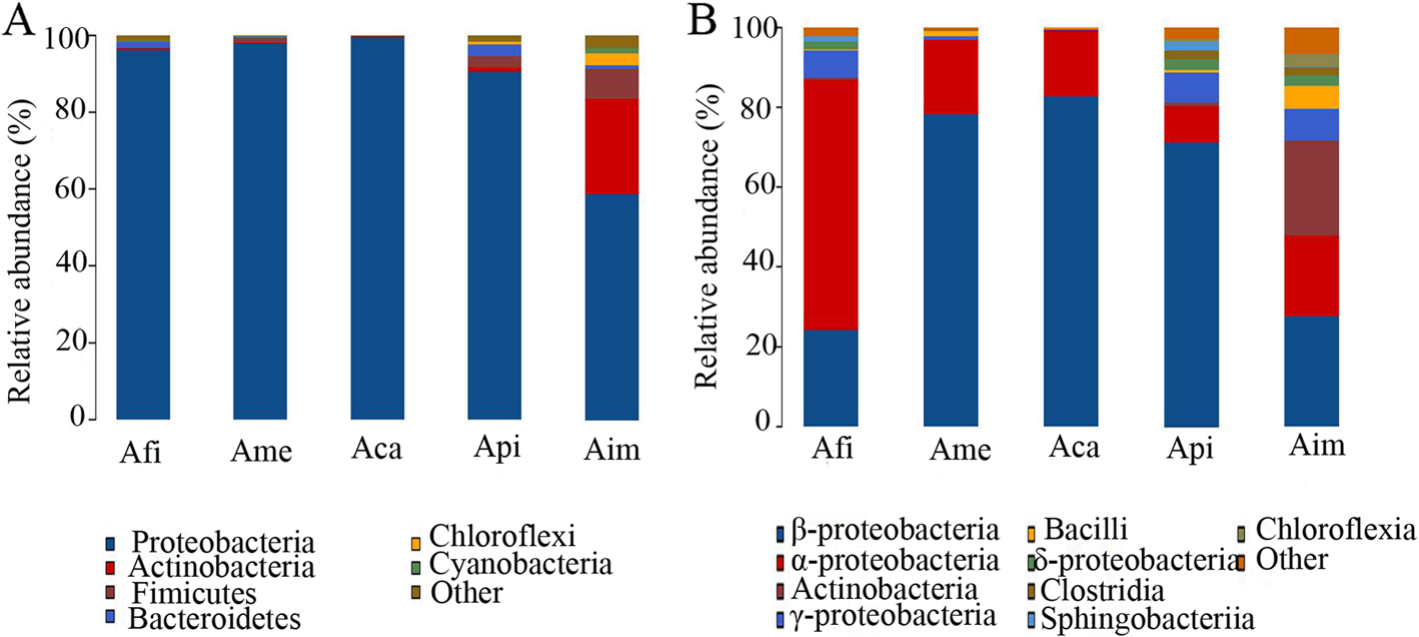
The relative abundance of bacteria across different species of *Azolla* at phylum and class level. Notes: **A** bacterial groups at the phylum level; **B** bacterial groups at the class level. The analysis method is based on the relative abundance of species at the phylum and class levels, using the ggplot2 package of the R (v3.6.0) software to perform histogram analysis of species composition. Species with a relative abundance of less than 1% are represented by other in the legend

The bacterial communities showed high diversity at the phylum level, totally up to22 bacterial phyla distributed among five species of *Azolla* (Supplementary Table 1). The relative abundance of Proteobacteria in Afi (92.96%), Ame (95.60%), and Aca (99.58%) was significantly higher than in Api (86.10%) and Aim (52.23%). While the relative abundance of Actinobacteria and Firmicutes in Afi (0.389%, 0.12%), Ame (0.22%, 1.13%), Aca (0.02%, 0.1%) was significantly lower than in Aim (24.33%, 7.02%) and Api (0.67%, 1.59%), respectively (Fig. 3A; Supplementary Table 1).

Bacterial communities are more diverse at the class level (Fig. 3B). Moreover, the bacterial groups and relative abundance of different species of *Azolla* are different. The bacterial community of Afi mainly include 62.63% α-Proteobacteria, 24.32% β-Proteobacteria, 7.06% γ-Proteobacteria, 2.10% δ- Proteobacteria, and 1.12% Sphingobacteriia. The bacterial community of Ame is mainly composed of 78.35% β-Proteobacteria, 18.58% α-Proteobacteria, and 1.11% Bacilli. And it is mainly composed of 82.83% β-Proteobacteria and 16.66% α-Proteobacteria of Aca. There are 71.18% β-Proteobacteria, 9.14% α-Proteobacteria, 7.46% γ-Proteobacteria, 2.67% δ-Proteobacteria, 2.25% Sphingobacteriia and 2.15% Clostridia of Api. While it is mainly composed of 27.64% β-Proteobacteria, 23.84% Actinobacteria, 20.17% α-Proteobacteria, 7.97% γ-Proteobacteria, 5.82% Bacilli, 2.98% Chloroflexia, 2.78% δ-Proteobacteria and 1.81% Clostridia of Aim (Fig. 3B; Supplementary Table 2).

**Fig. 4:**
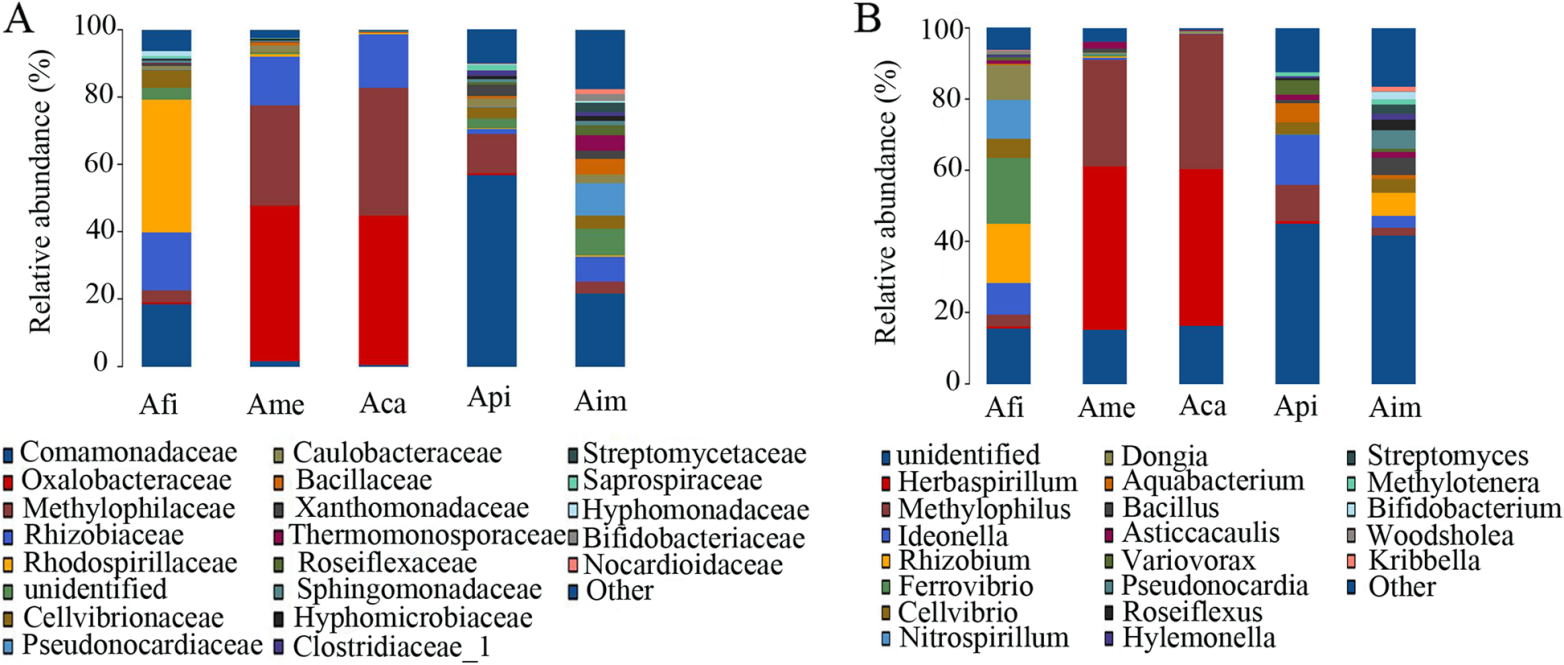
Bacterial community composition of different species of *Azolla* at family and genus level. Notes: **A** at the family level; **B** at the genus level. The analysis method is based on the relative abundance of species at the family and genus levels, using the ggplot2 package of the R (v3.6.0) software to perform histogram analysis of species composition. Species with a relative abundance of less than 1% are represented by other in the legend

The endophytic bacterial community composition of the five species of *Azolla* was different at the family level (Fig. 4A; Supplementary Table 3). In detail, the bacteria inhabiting Aca and Ame were relatively similar, and the bacterial diversity of these two *Azolla* was significantly lower than that of the other three. The family of Oxalobacteraceae belonged to the order of Burkholderiales was the predominant bacterial group in Ame (46.00%) and Aca (44.30%), however, it was less than 1% in the other three species. The family of Comamonadaceae was the dominant group in Api, with a relative abundance of 57.00%. While it accounted for 18.56% and 21.70% of the total bacteria of Afi and Aim, respectively. The relative abundances of Methylophilaceae in Ame and Aca were 30.00% and 38.00%, respectively, but their relative abundances in Api was 11.30%, and it was both less than 5.00% in Afi and Aim. The relative abundance of Rhizobiaceae was 17.17% in Afi, 14.27%, and 16.03% in Ame and Aca, respectively, while it was only 7.44% and 1.42% in Aim and Api, respectively. The most abundant bacterial group was Rhodospirillaceae in Afi (39.61%), but it was extremely low in the other four species of *Azolla*.

Further analysis revealed that endophytic bacterial communities showed high diversity and significant differences at the genus level among different species of *Azolla* (Fig. 4B; Supplementary Table 4). The bacterial communities of Ame and Aca were mainly composed of the genera of *Herbaspirillum* (45.91%, 44.08%) and *Methylophilus* (29.97, 37.96%), however, they were less than 5.00% in Afi and Aim. The relative abundance of *Ideonella* in Afi, Api, and Aim were 8.87%, 14.00%, and 3.43%, respectively, while it was less than 1.00% in both Ame and Aca. The genus of *Ferrovibrio* was the most abundant bacterial group in Afi (18.53%) and it was less than 1.00% in the other four species of *Azolla*. The genus of *Rhizobium* was the second abundant in Afi (16.68%), and it was 6.49% in Aim, but it was less than 1.00% in others. The relative abundance of the genera of *Nitrospirillum* and *Dongia* in Afi was 10.92% and 9.72%, respectively, but it was less than 1.00% in others. The relative abundance of *Cellvibrio* in Afi, Aim, and Api was 5.40%, 3.79%, and 3.31%, respectively, but less than 1.00% in Ame and Aca. *Bacillus*, *Asticcacaulis*, *Variovorax* were specific taxa in Aim, however, their relative abundances were relatively low. These results showed that different species of *Azolla* have an enrichment preference for the endophytic bacterial community.

### Drivers of variation in endophytic bacterial community composition in *Azolla*

**Fig. 5:**
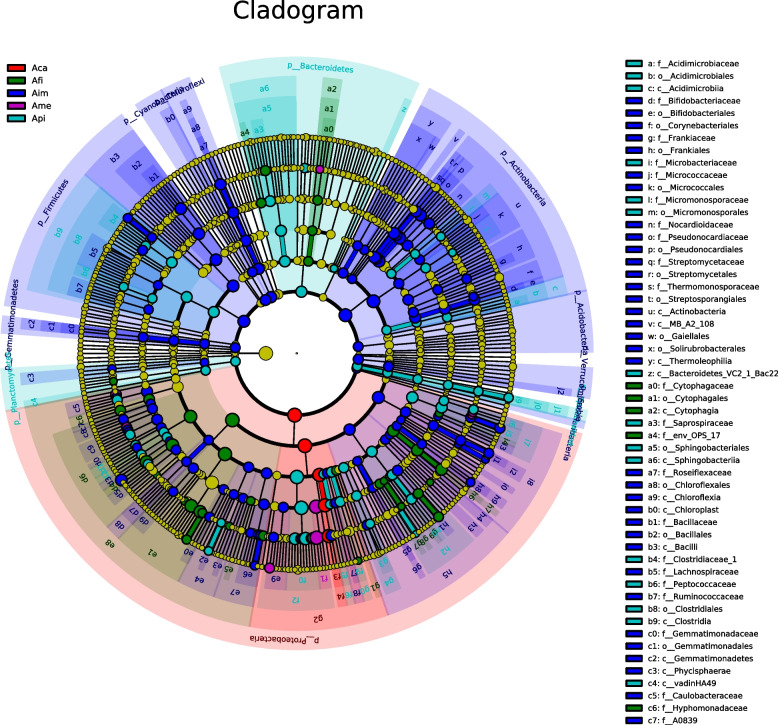
Identification of endophytic bacterial taxa that accounted for the greatest differences in different species of *Azolla*

To further explore the variation of the bacterial communities in different species of *Azolla*, the LEfSe analysis method was used to find bacteria with significant differences in abundance between samples (Fig. 5; Supplementary Fig. 1). In detail, at different taxa levels, more specific bacteria inhabiting in Aim than in other species of *Azolla*. The genus of *Methylophilus* was specific in Aca. The genera of *Herbaspirillum*, *Devosia* were specific in Ame. The phyla of Actinobacteria, Firmicutes, Chloroflexi, Cyanobacteria were more abundant in Aim compared to other species of *Azolla*. The family of Comamonadaceae was the richest group in Api. Moreover, the phyla of Bacteroidetes, Spirochaetae, and Planctomycetes exhibited a difference in relative abundance in Api compared to other *Azolla*. The class of Alphaproteobacteria was the most abundant in Afi, especially the genera of *Ferrovibrio*, *Rhizobium*, and *Nitrospirillum* (Fig. 5; Supplementary Fig. 1).

Circles radiating from inside to outside represent classification levels from phylum to genus. Each small circle at different taxonomic levels represents a classification at that level, with the small circle diameter size being proportional to the relative abundance size. Coloring principle: species with no significant differences were colored to yellow, and different species biomarker follows the group. Red nodes indicate microbial taxa that play an important role in the red group, green nodes indicate microbial taxa that play an important role in the green group, and other circle colors mean the same. The species names indicated by the English letters in the cladogram are shown in the legend to the right.

## Discussion

Various bacterial communities inhabiting the rhizosphere and phyllosphere of plants may have positive and negative effects on plant productivity and health and have received extensive attention in recent years [2, 9]. Most plants harbor diverse bacterial communities that help maintain plant–microbe balance and protect plants from fungi and Oomycota [29]. Plant-associated bacteria are also key players in global biogeochemical cycles, especially in soil nutrient cycling and greenhouse gas emissions [1]. The nitrogen-fixing cyanobacteria *Nostoc azollae* was considered as a mutually beneficial symbiotic bacteria of the water fern *Azolla*, that allows the plant to grow in nitrogen-deficient environments and make significant environmental benefits. Recent studies have shown that the phylogeny of *Azolla* with symbiotic cyanobacteria largely formed a co-evolutionary pattern [30]. In addition, diverse bacterial communities colonizing *Azolla* may play a key role in maintaining the homeostasis of the symbiont. Previous studies have shown that *Azolla* with symbiotic bacteria has stronger adaptability than that without bacteria [31]. However, as far as we know, the endophytic bacterial community composition inhabiting the phyllosphere of different species of *Azolla* has not yet been fully understood. According to this study, we detected and compared the diversity and composition of endophytic bacterial communities inhabiting the phyllosphere of different species of *Azolla* collected from different countries by Illumina high-throughput sequencing.

In the present study, we found that the endophytic bacterial community’s composition and diversity were complex and different among different species of *Azolla*. In detail, based on the OTU results and the α indices, the bacterial diversity and richness were ordered as Aim > Api > Afi > Ame > Aca (Table 1; Fig. 1; Table 2). In addition, based on the PCoA analysis, with the *Euazolla* subgenus, Ame and Aca shared most of the bacterial groups yet the bacterial community of Afi was in a relatively independent group. However, the bacterial communities were relatively close between Api and Aim, the two members of the *Rhizosperma* subgenus (Fig. 2). Further analysis via the RDP Classifier tool showed that the bacterial communities of *Azolla* cultivated in paddy soil medium were mainly composed of the phylum of Proteobacteria, followed by Actinobacteria, Firmicutes, Bacteroides, Chloroflexi and Cyanobacteria. In detail, the relative abundance of Proteobacteria accounted for nearly 50% in Aim and over 80% in the other four species of *Azolla* (Fig. 3). The recruitment of bacterial groups displayed distinct differences in different *Azolla* species (Fig. 3; Fig. 4), especially the bacterial community inhabiting the Aim contained the most significant different types of bacteria (Fig. 5). The phylum Proteobacteria is the most abundant bacterial group in different Azolla species cultured under the same conditons, indicating that the plant host controls the assembly of endophytic bacterial communities. And these differences may be due to the comprehensive effect of the difference in *Azolla* germplasm and their adaption to specific seasonal environmental factors.

The different bacteria inhabiting different species of *Azolla* may provide different auxiliary functions for hosts to adapt to the aquatic environment and perform ecological functions. For example, the endophytic *Rhizobium* was present in all species of *Azolla*, especially the highest relative abundance in Afi, and previous studies suggested that the main role of *Rhizobium* may be involved in the nitrogen cycle in the denitrification pathway [22]. *Rhizobium* is also advantageous nitrogen-fixing bacteria in the rice rhizosphere, therefore, *Azolla* may promote the enrichment of *Rhizobium* in the rice rhizosphere and thus promote the nitrogen cycle. As it was reported that some species of the *Herbaspirillum* genus were involved in the rice rhizosphere microbial reduction of the N_2_O process, which would greatly reduce greenhouse gases emission in paddy fields [32]. Therefore, the abundance of *Herbaspirillum* inhabiting Ame and Aca may participate in the nitrogen cycle process of the rice-*Azolla* co-cropping system and make a difference in greenhouse gas emissions. *Methylophilus* was the second abundant in Aca and Ame, and *Methylophilus* can oxidize the simplest hydronitrogen to produce microbial available organic matter, such as proteins, biopolymers, vitamins, and methanol [33, 34], and these organic matter may also be supplied to the plant host through specific pathways. Previous studies suggested that some bacteria of the *Ideonella* genus feed on PET plastic debris, and special enzymes in the bacteria decompose PET into environmentally harmless terephthalic acid and ethylene glycol [35, 36]. This means that *Azolla* could be useful for removing plastic contaminants from the environment and potentially opening new applications of Afi and Api for harboring a high relative abundance of *Ideonella*. As it was reported that *Ferrovibrio* was able to reduce bromate to bromide in water bodies [37], and bromate content is an important indicator to evaluate the health of water quality, therefore, Afi with high richness *Ferrovibrio* may play an important role in improving water health. All in all, these special dominant bacterial groups inhabiting the phyllosphere of different species of *Azolla* may play a key role in agricultural production or environmental management.

## Materials and methods

### Plant material

Five *Azolla* species served as the source of phyllosphere endophytic bacteria which have been collected from different countries and stored in the National Azolla Germplasm Resource Center, China. The information of tested samples are detailed in Table 3. The phenotype and parameters of each species of *Azolla* are showed in supplementary Fig. 2.

**Table 3:** Sampling information of five species of *Azolla*

Accession number	Species name	Abbreviation	Subgenera	Collection Origin
IRRI 1001	*Azolla filiculoides* lamarck	Afi	*Euazolla* subgenus	Germany, collected in 1979. Introduced from IRRI in 1979
IRRI 2002	*Azolla mexicana* Schlecht et cham	Ame		Guyana, collected in 1981. Introduced from IRRI in 1984
IRRI 3502	*Azolla caroliniana* Willd	Aca		Egypt, collected in 1983. Introduced from IRRI in 1989
NARC 500	*Azolla imbricata* (Roxb.) Nakais	Aim	*Rhizosperma*subgenus	China, collected in 1980. Saved in NARC
IRRI 7001	*Azolla pinnata* R.Brown	Api		Australia, collected in 1982. Introduced from IRRI in 1984

*IRRI* International Rice Research Institute, Philippines, *NARC* National Azolla Germplasm Resource Center, China

### Experimental settings

The experimental site was located in Fuzhou city, China (26°7’58”N, 119°19’58”E). Air-dried paddy soil was crushed, sieved to remove impurities, and then sterilized under the pressure of 0.105 MPa at 121℃ for 20 min. The sterilized soil was used as the cultivation substrate. Each small pond was added in sterile water and maintained a 5 cm soil layer and 7 cm water depth.

All plants with the same initial weight (1 g) were grown under the same conditions, and three repeats per species. All plants were cultured for one month before sampling. The cultivation substrate contained 1.80% organic matter, 0.11% total nitrogen, 0.66% full potassium, 0.17% whole phosphorus, 104.23 mg/kg alkali-hydrolyzable nitrogen, 10.43 mg/kg effective phosphorus, 30.30 mg/kg fast-acting potassium, pH 4.39. Five species of *Azolla* were cultivated under natural illumination, at 25℃, 80% relative humidity.

### Sample collection

When all of the *Azolla* samples covered the full water surface, healthy plants were then collected and washed with ddH_2_O to remove epiphytes and contaminants. Roots were removed from the plants to obtain frond-rich tissue. Sediment-free fronds were soaked in 70% ethanol for 40 s followed by a step in 2.5% sodium chloride for 10 min, and then immersed in sterile Milli-Q water three times to remove any excess of sodium chloride [38], then surface water dried with sterile filter paper and stored at -80℃. All samples were collected on November 17, 2020.

### Library preparation and amplicon sequencing

Total genomic DNA was extracted from each *Azolla* sample using a DNA Kit (Omega Bio-Tek, Norcross, GA, U.S.) following the manual. The purity and quality of the extracted genomic DNA were checked on 1% agarose gel and spectrophotometry. The extracted DNA was then used as templates for PCR amplification. The V5-V7 variable regions of the 16S ribosomal DNA gene of bacteria were amplified for assessing endophytic bacterial diversity using the universal primers 799F (5’-AACMGGATTAGATACCCKG-3’) and 1193R (5’-ACGTCATCCCCACCTTCC-3’) [39]. PCR amplifications were carried out on a Mastercycler Gradient (Eppendorf, Germany) using 25 μl reaction volumes, containing 12.5 μl KAPA 2G Robust Hot Start Ready Mix, 1 µl Forward Primer (5 µM), 1 µl Reverse Primer (5 µM), 5 µl DNA (total template quantity is 30 ng), and 5.5 µl H_2_O. The PCR reaction procedure consisted of an initial denaturation at 94℃ for 5 min; followed by 28 cycles consisting of denaturation at 94℃ for 30 s, annealing at 55℃ for 30 s, and extension at 72℃ for 60 s; and then final extension at 72℃ for 7 min. Three PCR products per sample were pooled to mitigate reaction-level PCR biases. PCR products were purified using a QIAquick Gel Extraction Kit (QIAGEN, Germany), quantified using Real-Time PCR. Deep sequencing was performed on the Miseq platform according to standard protocols at Allwegene Company (Beijing). After the run, image analysis, base calling, and error estimation were performed using Illumina Analysis Pipeline Version 2.6.

### Data analysis

The raw data were first screened and removed if they were shorter than 200 bp, had a low quality score (≤ 20), contained ambiguous bases, or did not exactly match primer sequences and barcode tags. Qualified reads were separated using the sample-specific barcode sequences and trimmed with Illumina Analysis Pipeline Version 2.6. High-quality sequences were then analyzed using QIIME (Version 1.8.0http://qiime.org/). The sequences were clustered into operational taxonomic units (OTU) at a similarity level of 97% performed by uparse, to generate rarefaction curves and to calculate the richness and diversity indices [40]. The Ribosomal Database Project (RDP) Classifier tool was used to classify all sequences into different taxonomic groups. The OTU data was used to calculate α and β diversity indices. For α diversity, the richness of each sample was evaluated using the PD whole tree and Chao1 index, and the diversity of each sample was estimated using the Shannon and Simpson index. To investigate the similarity among different samples, OTU information was analyzed using bray Curtis-based principal coordinate analysis (PCoA) [41]. To obtain the taxonomic information corresponding to each OTU, the representative sequences of OTUs were compared and analyzed by the RDP Classifier algorithm at each level (Phylum, class, family, genus) to annotate the specific information of the community, confidence threshold is 0.7 [42]. Furthermore, the LDA Effect Size (LEFSe) analysis was used to detect bacteria with significant differences in abundance among different species of *Azolla* [43]. All statistical analyses were performed in the R environment using the igraph package, psych package.

## Conclusions

Based on the Alpha index results showed that, considering the abundance and uniformity of bacterial community, the diversity ordered as Aim > Api > Afi > Ame > Aca. The phyllosphere endophytic bacterial communities of different species of *Azolla* cultivated in paddy soil were mainly composed of Proteobacteria, Actinobacteria, Firmicutes, Bacterioides, Chloroflexi and Cyanobacteria. The relative abundance of Proteobacteria accounts for nearly 60% in Aim, and over 95% in the other four *Azolla*. Ame and Aca shared most of the bacterial taxa. The cluster of bacterial communities in Api and Aim were similar at different taxonomic levels. The symbiotic bacterial community of Afi was relatively independent.

## Supplementary Information

**Additional file 1:** **Supplementary Table 1.** The relative abundance of bacteria across different species of *Azolla* at phylum level.

**Additional file 2:** **Supplementary Table 2.** The relative abundance of bacteria across different species of *Azolla* at class level.

**Additional file 3:** **Supplementary Table 3.** The relative abundance of bacteria at family level in different species of *Azolla.*

**Additional file 4:** **Supplementary Table 4.** The relative abundance of bacteria at genus level in different species of *Azolla.*

**Additional file 5. **

**Additional file 6:** **Supplementary Figure 2.** The phenotype and parameters of five species of *Azolla.*

## Data Availability

The sequence data generated and analyzed in this study are available at NCBI (https://www.ncbi.nlm.nih.gov) under accession numbers (PRJNA817019).

## References

[1] Turner TR, James EK, Poole PS (2013). The plant microbiome. Genome Biol.

[2] Trivedi P, Leach JE, Tringe SG, Sa TM, Singh BK (2020). Plant-microbiome interactions: from community assembly to plant health. Nat Rev Microbiol.

[3] Vandenkoornhuyse P, Quaiser A, Duhamel M, Van AL, Dufresne A (2015). The importance of the microbiome of the plant holobiont. New Phytol.

[4] Agler MT, Ruhe J, Kroll S, Morhenn C, Kim ST, Weigel D (2016). Microbial hub taxa link host and abiotic factors to plant microbiome variation. PLoS Biol.

[5] Lindow SE, Brandl MT (2003). Microbiology of the phyllosphere. Appl Environ Microbiol.

[6] Peiffer JA, Spor A, Koren O, Jin Z, Tringe SG, Dangl JL (2013). Diversity and heritability of the maize rhizosphere microbiome under field conditions. Proc Natl Acad Sci.

[7] Carbonnel S, Gutjahr C (2014). Control of arbuscular mycorrhiza development by nutrient signals. Front Plant Sci.

[8] Bulgarelli D, Garrido-Oter R, Münch PC, Weiman A, Dröge J, Pan Y (2015). Structure and function of the bacterial root microbiota in wild and domesticated barley. Cell Host Microbe.

[9] Chen SM, Waghmode TR, Sun R, Kuramae EE, Hu CS, Liu BB (2019). Root-associated microbiomes of wheat under the combined effect of plant development and nitrogen fertilization. Microbiome.

[10] Horton MW, Bodenhausen N, Beilsmith K, Meng DZ, Muegge BD, Subremanian S (2014). Genome-wide association study of *Arabidopsis thaliana’*s leaf microbial community. Nat Commun.

[11] Edwards J, Johnson C, Santos-Medellín C, Lurie E, Podishetty NK, Bhatnagar S (2015). Structure, variation, and assembly of the root-associated microbiomes of rice. Proc Natl Acad Sci.

[12] Latz MAC, Kerrn MH, Sørensen H, Collinge DB, Jensen B, Brown JKM (2021). Succession of the fungal endophytic microbiome of wheat is dependent on tissue-specific interactions between host genotype and environment. Sci Total Environ.

[13] Rodriguez PA, Rothballer M, Chowdhury SP, Nussbaumer T, Gutjahr C, Falter-Braun P (2019). Systems biology of plant-microbiome interactions. Mol Plant.

[14] Lumpkin TA, Plucknett DL (1980). *Azolla*: botany, physiology, and use as green manure. Econ Bot.

[15] Chen J, Xu GZ (2001). The progress in systematic studies of *Azolla*. Chinese Bulletin of Botany.

[16] Roy R, Reinders A, Ward JM, McDonald TR (2020). Understanding transport processes in lichen, *Azolla*-*cyanobacteria*, ectomycorrhiza, endomycorrhiza, and rhizobia-legume symbiotic interactions. F1000Research.

[17] Setiawati MR, Prayoga MK, Stöber S, Adinata K, Simarmata T (2020). Performance of rice paddy varieties under various organic soil fertility strategies. Open Agriculture.

[18] Yang GY, Ji HT, Sheng J, Zhang YF, Feng YF, Guo Z (2020). Combining *Azolla* and urease inhibitor to reduce ammonia volatilization and increase nitrogen use efficiency and grain yield of rice. Sci Total Environ.

[19] De AK, Ghosh A, Dolui D, Saha I, Adak MK (2020). 2,4-D hyper accumulation induced cellular responses of Azolla pinnata R Br to sustain herbicidal stress. Phyton-Int J Exp Bot.

[20] Novair SB, Hosseini HM, Etesami H, Razavipour T (2020). Rice straw and composted *Azolla* alter carbon and nitrogen mineralization and microbial activity of a paddy soil under drying–rewetting cycles. Appl Soil Ecol.

[21] Bianchi E, Biancalani A, Berardi C, Antal A, Fibbi D, Coppi A (2020). Improving the efficiency of wastewater treatment plants: Bio-removal of heavy-metals and pharmaceuticals by *Azolla filiculoides* and *Lemna minuta*. Sci Total Environ.

[22] Forni C, Riov J, Caiola MG, Tel-Or E (1992). Indole-3-acetic acid (IAA) production by *Arthrobacter* species isolated from *Azolla*. J Gen Microbiol.

[23] Serrano R, Carrapico F, Vidal R (1999). The presence of lectins in bacteria associated with the *Azolla*-*Anabaena* symbiosis. Symbiosis.

[24] Zheng SP, Chen B, Guan X, Zheng WW (2008). Diversity analysis of endophytic bacteria within *Azolla microphylla* using PCR-DGGE and electron microscopy. J Agric Biotech.

[25] Zheng SP, Chen B, Wang J, Lu PJ (2012). T-RFLP analysis on diversity of endophytic bacteria in *Azolla*
*microphylla*. J Anhui Agric Sci.

[26] Dijkhuizen LW, Brouwer P, Bolhuis H, Reichart GJ, Koppers N, Huettel B (2018). Is there foul play in the leaf pocket? The metagenome of floating fern *Azolla* reveals endophytes that do not fix N_2_ but may denitrify. New Phytol.

[27] Chen J, Zheng WW, Zheng YP, Chen B, Zheng SP, Zhu BY (2019). Discovery of eno-fungi and archaea within water fern *Azolla microphylla* and their community analyses based on high throughput sequencing. J Agric Biotech.

[28] Carrapico F (2002). The *Azolla*-*Anabaena*-bacteria system as a natural microcosm. Proc SPIE.

[29] Duran P, Thiergart T, Garrido-Oter R, Agler M, Kemen E, Schulze-Lefert S (2018). Microbial interkingdom interactions in roots promote *Arabidopsis* survival. Cell.

[30] Li FW, Brofuwer P, Carretero-Paulet L, Cheng SF, Vries JD, Delaux PM (2018). Fern genomes elucidate land plant evolution and cyanobacterial symbioses. Nat Plants.

[31] Lin C, Watanable I (1992). Effects of *Anabaena **Azollae* on the tolerance of *Azolla* to high temperature[J]. J Fujian Acad Agric Sci.

[32] Ishii S, Ohno H, Tsuboi M, Otsuka S, Senoo K (2011). Identification and isolation of active N_2_O reducers in rice paddy soil. ISME J.

[33] Strong PJ, Xie SH, Clarke WP (2015). Methane as a resource: can the methanotrophs add value?. Environ Sci Technol.

[34] Tsapekos P, Zhu XY, Pallis E, Angelidaki I (2020). Proteinaceous methanotrophs for feed additive using biowaste as carbon and nutrients source. Biores Technol.

[35] Tournier V, Topham CM, Gilles A, David B, Folgoas C, Moya-Leclair E (2020). An engineered PET depolymerase to break down and recycle plastic bottles. Nature.

[36] Yoshida S, Hiraga K, Takehana T, Taniguchi I, Yamaji H, Maeda Y (2016). A bacterium that degrades and assimilates poly (ethylene terephthalate). Science.

[37] Liu CS, Li W, Liu LH, Yu HT, Liu F, Lee DJ (2020). Autotrophic induced heterotrophic bioreduction of bromate in use of elemental sulfur or zerovalent iron as electron donor. Biores Technol.

[38] Tarquinio F, Attlan O, Vanderklift MA, Berry O, Bissett A (2021). Distinct endophytic bacterial communities inhabiting seagrass seeds. Front Microbiol.

[39] Bodenhausen N, Horton MW, Bergelson J (2013). Bacterial communities associated with the leaves and the roots of *Arabidopsis thaliana*. PLoS ONE.

[40] Edgar RC (2013). UPARSE: highly accurate OTU sequences from microbial amplicon reads. Nat Methods.

[41] Wang Y, Sheng HF, He Y, Wu JY, Jiang YX, Tam NFY (2012). Comparison of the levels of bacterial diversity in freshwater, intertidal wetland, and marine sediments by using millions of illumina tags. Appl Environ Microbiol.

[42] Jiang XT, Peng X, Deng GH, Sheng HF, Wang Y, Zhou HW (2013). Illumina sequencing of 16S rRNA tag revealed spatial variations of bacterial communities in a mangrove wetland. Microb Ecol.

[43] Segata N, Izard J, Waldron L, Gevers D, Miropolsky L, Garrett W (2011). Metagenomic biomarker discovery and explanation. Genome Biol.

