# Supplementary material for: Comparative analysis of the endophytic bacteria inhabiting the phyllosphere of aquatic fern Azolla species by high-throughput sequencing
Source: BMC Microbiol. 2022 Oct 11;22:246. doi: 10.1186/s12866-022-02639-2 (PMC9552495; doi:10.1186/s12866-022-02639-2)
Supplement: Supplementary file 1 — Additional file 1: Supplementary Table 1. The relative abundance of bacteria across different species of Azolla at phylum level. [file 12866_2022_2639_MOESM1_ESM.docx]

Supplementary Table 1 The relative abundance of bacteria across different species of *Azolla* at phylum level

| Phylum | Afi  Mean±SEM, n=3 | Ame  Mean±SEM, n=3 | Aca  Mean±SEM, n=3 | Api  Mean±SEM, n=3 | Aim  Mean±SEM, n=3 |
| --- | --- | --- | --- | --- | --- |
| Ignavibacteriae | 0.00±0.00 | 0.00±0.00 | 0.00±0.00 | 0.00±0.00 | 0.07±0.07 |
| Planctomycetes | 0.18±0.03 | 0.00±0.00 | 0.00±0.00 | 0.31±0.04 | 0.23±0.03 |
| SBR1093 | 0.00±0.00 | 0.00±0.00 | 0.00±0.00 | 0.00±0.00 | 0.00±0.00 |
| Verrucomicrobia | 0.50±0.09 | 0.00±0.00 | 0.00±0.00 | 0.42±0.10 | 0.684±0.16 |
| WS6 | 0.00±0.00 | 0.00±0.00 | 0.00±0.00 | 0.00±0.00 | 0.00±0.00 |
| Saccharibacteria | 0.00±0.00 | 0.00±0.00 | 0.00±0.00 | 0.02±0.01 | 0.30±0.15 |
| Fusobacteria | 0.00±0.00 | 0.00±0.00 | 0.00±0.00 | 0.00±0.00 | 0.03±0.02 |
| Proteobacteria | 96.15±0.52 | 98.00±0.39 | 99.68±0.15 | 90.59±1.93 | 58.64±0.31 |
| Bacteroidetes | 1.69±0.21 | 0.33±0.16 | 0.08±0.03 | 2.96±0.11 | 1.07±0.21 |
| Firmicutes | 0.22±0.04 | 1.19±0.37 | 0.18±0.13 | 3.00±2.30 | 7.68±0.57 |
| Omnitrophica | 0.00±0.00 | 0.00±0.00 | 0.00±0.00 | 0.00±0.00 | 0.00±0.00 |
| Spirochaetae | 0.00±0.00 | 0.00±0.00 | 0.00±0.00 | 0.29±0.06 | 0.24±0.05 |
| Gemmatimonadetes | 0.15±0.03 | 0.00±0.00 | 0.00±0.00 | 0.06±0.03 | 0.46±0.04 |
| Acidobacteria | 0.23±0.09 | 0.04±0.04 | 0.00±0.00 | 0.13±0.00 | 0.45±0.07 |
| Armatimonadetes | 0.00±0.00 | 0.00±0.00 | 0.00±0.00 | 0.00±0.00 | 0.03±0.02 |
| WS2 | 0.00±0.00 | 0.00±0.00 | 0.00±0.00 | 0.00±0.00 | 0.00±0.00 |
| Parcubacteria | 0.00±0.00 | 0.00±0.00 | 0.00±0.00 | 0.00±0.00 | 0.00±0.00 |
| Nitrospirae | 0.00±0.00 | 0.00±0.00 | 0.00±0.00 | 0.00±0.00 | 0.13±0.07 |
| Chloroflexi | 0.06±0.02 | 0.00±0.00 | 0.00±0.00 | 0.61±0.39 | 3.10±1.67 |
| Tectomicrobia | 0.00±0.00 | 0.00±0.00 | 0.00±0.00 | 0.00±0.00 | 0.02±0.02 |
| Cyanobacteria | 0.01±0.01 | 0.05±0.02 | 0.00±0.00 | 0.15±0.14 | 1.44±0.25 |
| TM6_Dependentiae | 0.00±0.00 | 0.00±0.00 | 0.00±0.00 | 0.01±0.01 | 0.15±0.08 |
| Actinobacteria | 0.47±0.14 | 0.21±0.16 | 0.03±0.01 | 1.14±0.23 | 24.94±2.19 |
| Chlorobi | 0.00±0.00 | 0.00±0.00 | 0.00±0.00 | 0.11±0.02 | 0.03±0.02 |
| Elusimicrobia | 0.00±0.00 | 0.00±0.00 | 0.00±0.00 | 0.00±0.00 | 0.04±0.03 |
| unidentified | 0.01±0.01 | 0.03±0.01 | 0.00±0.00 | 0.06±0.03 | 0.02±0.02 |
| Deinococcus-Thermus | 0.01±0.01 | 0.12±0.12 | 0.00±0.00 | 0.01±0.01 | 0.04±0.04 |
| Chlamydiae | 0.31±0.18 | 0.10±0.00 | 0.00±0.00 | 0.12±0.06 | 0.21±0.03 |
