# Supplementary material for: Comparative analysis of the endophytic bacteria inhabiting the phyllosphere of aquatic fern Azolla species by high-throughput sequencing
Source: BMC Microbiol. 2022 Oct 11;22:246. doi: 10.1186/s12866-022-02639-2 (PMC9552495; doi:10.1186/s12866-022-02639-2)
Supplement: Supplementary file 2 — Additional file 2: Supplementary Table 2. The relative abundance of bacteria across different species of Azolla at class level. [file 12866_2022_2639_MOESM2_ESM.docx]

Supplementary Table 2 The relative abundance of bacteria across different species of *Azolla* at class level

| Class | Afi  Mean±SEM, n=3 | Ame  Mean±SEM, n=3 | Aca  Mean±SEM, n=3 | Api  Mean±SEM, n=3 | Aim  Mean±SEM, n=3 |
| --- | --- | --- | --- | --- | --- |
| Betaproteobacteria | 24.32±1.22 | 78.35±1.81 | 82.83±1.10 | 71.18±0.35 | 27.64±0.22 |
| Alphaproteobacteria | 62.63±2.09 | 18.58±1.98 | 16.66±0.98 | 9.14±0.58 | 20.17±0.32 |
| Actinobacteria | 0.39±0.14 | 0.20±0.15 | 0.023±0.01 | 0.86±0.22 | 23.84±2.10 |
| Gammaproteobacteria | 7.06±0.66 | 0.90±0.28 | 0.18±0.06 | 7.46±1.81 | 7.97±0.51 |
| Bacilli | 0.055±0.01 | 1.12±0.38 | 0.18±0.13 | 0.85±0.56 | 5.82±0.39 |
| Deltaproteobacteria | 2.10±0.27 | 0.16±0.01 | 0.016±0.02 | 2.67±0.37 | 2.78±0.18 |
| Clostridia | 0.16±0.03 | 0.08±0.05 | 0.01±0.00 | 2.15±1.74 | 1.81±0.23 |
| Sphingobacteriia | 1.12±0.19 | 0.12±0.11 | 0.03±0.01 | 2.25±0.10 | 0.37±0.07 |
| Chloroflexia | 0.06±0.02 | 0.00±0.00 | 0.00±0.00 | 0.58±0.37 | 2.98±1.57 |
| other | 2.11±0.29 | 0.48±0.17 | 0.09±0.02 | 2.87±0.17 | 6.601±0.57 |
