# Supplementary material for: Comparative analysis of the endophytic bacteria inhabiting the phyllosphere of aquatic fern Azolla species by high-throughput sequencing
Source: BMC Microbiol. 2022 Oct 11;22:246. doi: 10.1186/s12866-022-02639-2 (PMC9552495; doi:10.1186/s12866-022-02639-2)
Supplement: Supplementary file 3 — Additional file 3: Supplementary Table 3. The relative abundance of bacteria at family level in different species of Azolla. [file 12866_2022_2639_MOESM3_ESM.docx]

Supplementary Table 3 The relative abundance of bacteria at family level in different species of *Azolla*

| Family | Afi  Mean±SEM, n=3 | Ame  Mean±SEM, n=3 | Aca  Mean±SEM, n=3 | Api  Mean±SEM, n=3 | Aim  Mean±SEM, n=3 |
| --- | --- | --- | --- | --- | --- |
| Comamonadaceae | 18.56±0.96 | 1.76±0.51 | 0.61±0.26 | 56.81±3.77 | 21.56±0.30 |
| Oxalobacteraceae | 0.60±0.16 | 45.94±2.40 | 44.10±1.19 | 0.69±0.67 | 0.153±0.08 |
| Methylophilaceae | 3.38±0.17 | 29.98±3.41 | 37.97±1.52 | 11.49±3.72 | 3.47±0.29 |
| Rhizobiaceae | 17.18±2.64 | 14.27±1.01 | 16.03±0.68 | 1.42±0.19 | 7.44±0.74 |
| Rhodospirillaceae | 39.61±3.98 | 0.85±0.50 | 0.35±0.32 | 0.36±0.06 | 0.23±0.07 |
| unidentified | 3.42±0.36 | 0.39±0.19 | 0.076±0.03 | 2.97±0.18 | 8.15±0.21 |
| Cellvibrionaceae | 5.41±0.76 | 0.14±0.07 | 0.078±0.06 | 3.31±0.98 | 3.79±0.17 |
| Pseudonocardiaceae | 0.16±0.12 | 0.04±0.00 | 0.00±0.00 | 0.14±0.05 | 9.59±1.15 |
| Caulobacteraceae | 0.89±0.05 | 2.00±0.87 | 0.054±0.02 | 2.40±0.43 | 2.53±0.19 |
| Bacillaceae | 0.05±0.01 | 1.04±0.39 | 0.17±0.13 | 0.82±0.55 | 4.80±0.29 |
| Xanthomonadaceae | 0.88±0.15 | 0.084±0.05 | 0.06±0.02 | 3.44±0.79 | 2.36±0.24 |
| Thermomonosporaceae | 0.00±0.00 | 0.00±0.00 | 0.00±0.00 | 0.00±0.00 | 4.59±1.06 |
| Roseiflexaceae | 0.06±0.02 | 0.00±0.00 | 0.00±0.00 | 0.58±0.37 | 2.98±1.57 |
| Sphingomonadaceae | 0.65±0.09 | 0.46±0.06 | 0.13±0.11 | 1.03±0.13 | 1.30±0.20 |
| Hyphomicrobiaceae | 0.53±0.12 | 0.61±0.23 | 0.01±0.01 | 0.86±0.05 | 1.48±0.10 |
| Clostridiaceae_1 | 0.11±0.01 | 0.01±0.01 | 0.01±0.00 | 1.65±1.43 | 1.09±0.05 |
| Streptococcaceae | 0.00±0.00 | 0.00±0.00 | 0.00±0.00 | 0.00±0.00 | 0.34±0.07 |
| Saprospiraceae | 0.71±0.14 | 0.01±0.00 | 0.01±0.01 | 1.70±0.11 | 0.16±0.01 |
| Hyphomonadaceae | 1.56±0.29 | 0.11±0.08 | 0.01±0.00 | 0.23±0.05 | 0.52±0.12 |
| Bifidobacteriaceae | 0.02±0.01 | 0.00±0.00 | 0.00±0.00 | 0.08±0.01 | 1.97±0.45 |
| Nocardioidaceae | 0.01±0.00 | 0.00±0.00 | 0.00±0.00 | 0.03±0.03 | 1.53±0.11 |
| other | 6.3±0.83 | 2.36±0.51 | 0.34±0.07 | 10.11±0.46 | 20.00±1.43 |
