# Supplementary material for: Comparative analysis of the endophytic bacteria inhabiting the phyllosphere of aquatic fern Azolla species by high-throughput sequencing
Source: BMC Microbiol. 2022 Oct 11;22:246. doi: 10.1186/s12866-022-02639-2 (PMC9552495; doi:10.1186/s12866-022-02639-2)
Supplement: Supplementary file 4 — Additional file 4: Supplementary Table 4. The relative abundance of bacteria at genus level in different species of Azolla. [file 12866_2022_2639_MOESM4_ESM.docx]

Supplementary Table 4 The relative abundance of bacteria at genus level in different species of *Azolla*

| Genus | Afi  Mean±SEM, n=3 | Ame  Mean±SEM, n=3 | Aca  Mean±SEM, n=3 | Api  Mean±SEM, n=3 | Aim  Mean±SEM, n=3 |
| --- | --- | --- | --- | --- | --- |
| unidentified | 15.47±1.32 | 15.19±0.80 | 16.29±0.69 | 44.92±7.42 | 41.63±1.65 |
| Herbaspirillum | 0.596±0.16 | 45.92±2.38 | 44.08±1.21 | 0.68±0.67 | 0.03±0.03 |
| Methylophilus | 3.33±0.16 | 29.98±3.41 | 37.96±1.52 | 10.37±3.71 | 2.17±0.20 |
| Ideonella | 8.87±0.61 | 0.55±0.23 | 0.15±0.11 | 14.00±0.39 | 3.44±0.25 |
| Rhizobium | 16.68±2.57 | 0.48±0.19 | 0.19±0.18 | 0.10±0.02 | 6.50±0.36 |
| Ferrovibrio | 18.54±2.16 | 0.39±0.23 | 0.20±0.19 | 0.09±0.03 | 0.00±0.00 |
| Cellvibrio | 5.40±0.76 | 0.19±0.07 | 0.08±0.06 | 3.31±0.98 | 3.79±0.17 |
| Nitrospirillum | 10.92±2.51 | 0.24±0.15 | 0.08±0.07 | 0.01±0.01 | 0.00±0.00 |
| Dongia | 9.73±2.56 | 0.22±0.12 | 0.07±0.06 | 0.02±0.02 | 0.04±0.04 |
| Aquabacterium | 0.54±0.04 | 0.16±0.05 | 0.08±0.04 | 5.41±2.35 | 1.11±0.11 |
| Bacillus | 0.05±0.01 | 1.00±0.40 | 0.17±0.13 | 0.82±0.55 | 4.77±0.31 |
| Asticcacaulis | 0.77±0.07 | 1.85±0.79 | 0.02±0.02 | 1.64±0.25 | 1.78±0.15 |
| Variovorax | 0.95±0.09 | 0.05±0.02 | 0.03±0.02 | 3.93±0.21 | 0.93±0.12 |
| Pseudomonas | 0.05±0.02 | 0.32±0.07 | 0.01±0.01 | 0.02±0.01 | 0.76±0.27 |
| Roseiflexus | 0.06±0.02 | 0.00±0.00 | 0.00±0.00 | 0.58±0.37 | 2.99±1.57 |
| Hylemonella | 0.61±0.05 | 0.14±0.07 | 0.05±0.03 | 0.44±0.03 | 1.64±0.19 |
| Streptococcus | 0.00±0.00 | 0.00±0.00 | 0.00±0.00 | 0.00±0.00 | 0.34±0.07 |
| Methylotenera | 0.05±0.00 | 0.01±0.00 | 0.04±0.00 | 1.12±0.02 | 1.30±0.11 |
| Bifidobacterium | 0.02±0.01 | 0.00±0.00 | 0.00±0.00 | 0.01±0.01 | 1.97±0.45 |
| Woodsholea | 1.26±0.01 | 0.03±0.01 | 0.01±0.01 | 0.05±0.02 | 0.35±0.08 |
| Kribbella | 0.00±0.00 | 0.00±0.00 | 0.00±0.00 | 0.00±0.00 | 1.31±0.14 |
| other | 6.17±0.74 | 3.38±0.27 | 0.52±0.22 | 12.50±1.64 | 23.20±0.55 |
