# Supplementary figures and images for: Comparative analysis of the endophytic bacteria inhabiting the phyllosphere of aquatic fern Azolla species by high-throughput sequencing
Source: BMC Microbiol. 2022 Oct 11;22:246. doi: 10.1186/s12866-022-02639-2 (PMC9552495; doi:10.1186/s12866-022-02639-2)

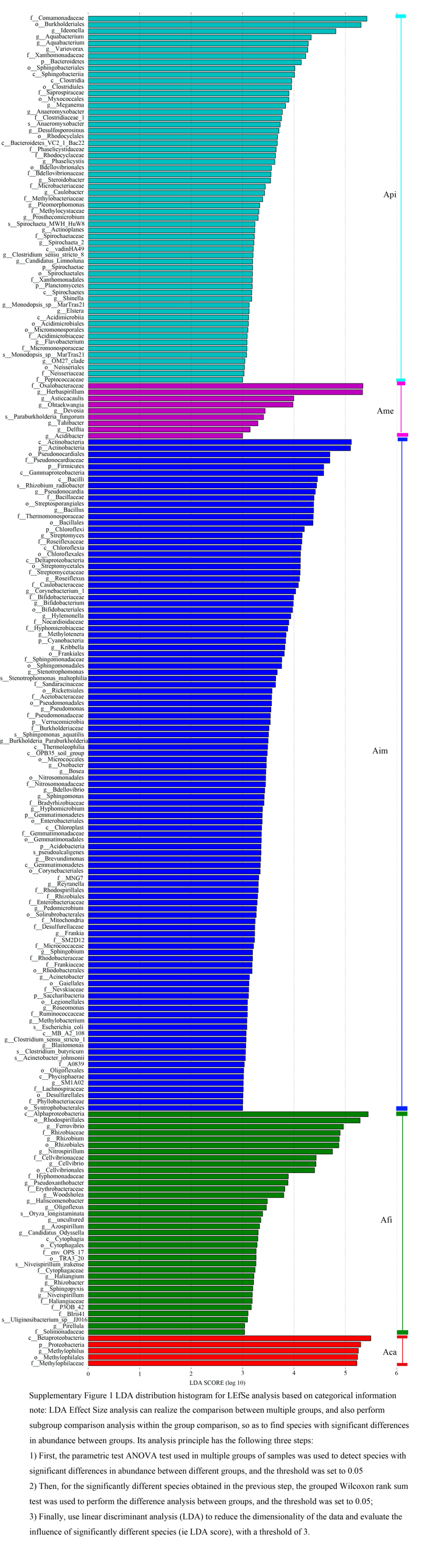

Supplement: Supplementary file 5 — Additional file 5. [file 12866_2022_2639_MOESM5_ESM.tif]

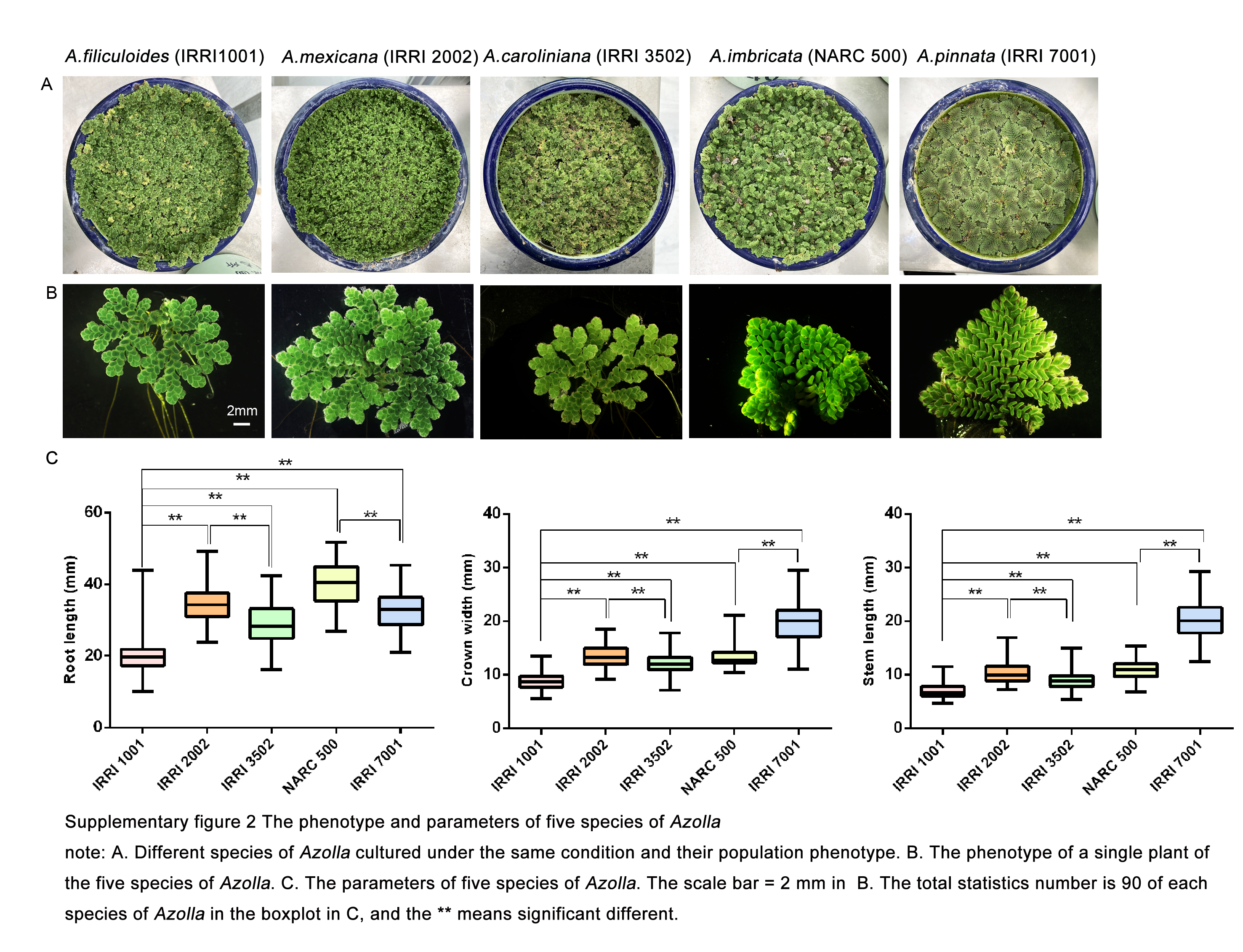

Supplement: Supplementary file 6 — Additional file 6: Supplementary Figure 2. The phenotype and parameters of five species of Azolla. [file 12866_2022_2639_MOESM6_ESM.tif]
